# Obituary: Paul Smith (1960–2025)

**DOI:** 10.3389/fneur.2026.1790952

**Published:** 2026-02-20

**Authors:** Ian S. Curthoys

**Affiliations:** Vestibular Research Laboratory, School of Psychology, Faculty of Science, University of Sydney, Sydney, NSW, Australia

**Keywords:** Alzheimer's disease, hippocampus, Parkinson's disease, tinnitus, vestibular compensation

## Introduction

**Figure 1:**
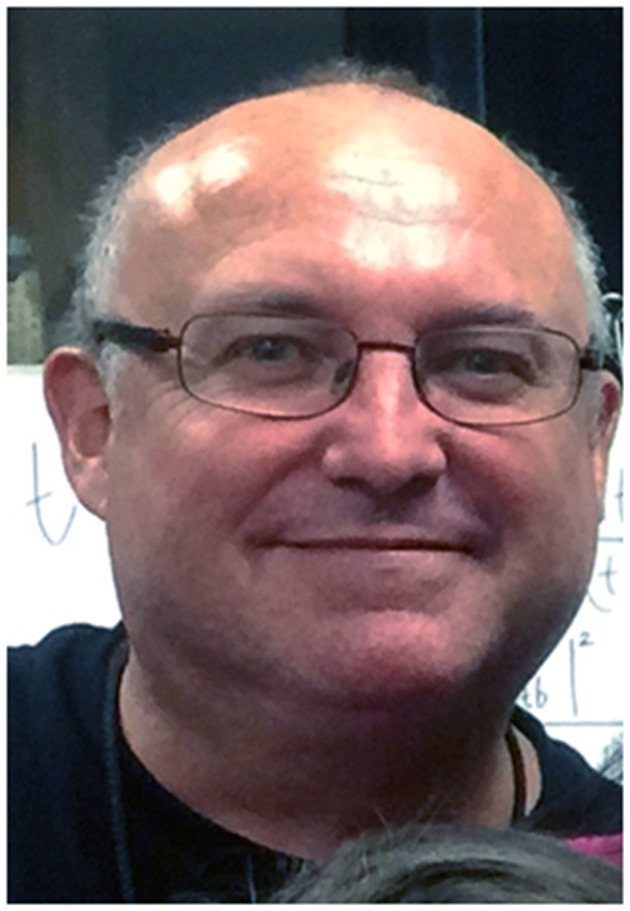
Paul Smith in 2018.

On 16 December 2025, Paul Frederick Smith passed away in his sleep at his home in Karitane near Dunedin, New Zealand, aged 65. At the time of his death, he was Professor of Neuropharmacology, (Personal Chair) in the Department of Pharmacology and Toxicology at the University of Otago, Dunedin, New Zealand. He was a major contributor in the field of vestibular knowledge, and his sudden death is a great loss not only to his collaborator and wife, Dr Cynthia Darlington, but also to his many colleagues, students and friends, to the wider scientific community and to the field of vestibular knowledge. Figure 1 is a photo of Paul Smith in 2018.

Paul was born on 5 December 1960 and graduated with a PhD from Sydney University in 1987. According to ResearchGate (https://www.researchgate.net/profile/Paul-Smith-77), in October 1987 Paul was appointed as a lecturer at University of Otago's Department of Psychology. There he and Cynthia Darlington won a grant from the New Zealand Medical Research Council and resumed their research on neural mechanisms of vestibular compensation.

In 1995 he was appointed as Associate Professor in the Department of Pharmacology and Toxicology at Otago University where he stayed for the rest of his career. He was promoted to professor (personal chair) in 1999. In 2014 Otago University awarded Paul a DSc in Vestibular Neuroscience. In 2013 he was awarded a Master of Applied Statistics with Honors from Massey University which led to his appointment as a Chartered Statistician by the Royal Statistical Society UK. So, he was an experimentalist but also a qualified statistician and he demanded excellence in experimental design and statistics in the very many papers that he published and reviewed.

The topic of his PhD was the change in neural activity in the vestibular nuclei occurring after unilateral vestibular loss—a process called vestibular compensation. He recorded single neurons in guinea pigs at various stages after unilateral vestibular loss and demonstrated the major imbalance in neural activity between the two vestibular nuclei shortly after unilateral vestibular loss and its progressive rebalancing over the next year. He used extracellular single neuron recording to record from an enormous number of single semicircular canal neurons in guinea pigs before and during vestibular compensation documenting the changes in resting activity and response to stimulation and so writing out many basic neural facts about vestibular compensation which still stand. The diminution of neural resting activity and reduced responses of neurons in the ipsilesional vestibular nuclei and the corresponding increase in activity of neurons in the contralesional vestibular nucleus. He chronicled the progressive return of neural activity and re-balancing of activity between the two vestibular nuclei corresponding to the progressive diminution of behavioral symptoms in guinea pigs and most probably in human patients after unilateral vestibular loss as in vestibular neuritis. An objective indicator of his excellence is the fact the research he conducted for his PhD has been so widely cited over the last 40 years.

Paul published his PhD research in landmark papers linking vestibular basic science to human clinical observations (1–3). Such links became a pattern for much of his research for the rest of his career- relating basic vestibular science to the clinic. These papers have served as the foundation for understanding the basic neural processes occurring during compensation, even down to the level of the molecular mechanisms responsible for the neural changes of compensation and more recently the neural basis of tinnitus. He had an abiding interest in the pharmacology of the vestibular system and the intracellular changes which are responsible for the restoration of activity in vestibular compensation. He also studied such diverse areas as the neuroprotective properties of Ginko Biloba and cannabis.

While he maintained a strong interest in the vestibular pathways to the oculomotor and postural control systems, his research extended far into these areas and beyond them. With his colleagues in Europe, he showed the role of vestibular input in human cognition. Recently he has focussed on the causes of tinnitus.

As I have noted, a major part of his medal-winning PhD was a review of the literature on vestibular compensation. This was comprehensive, critical but fair and he used his skill in reviewing large literatures in writing major reviews throughout his career. These have introduced many people to the vestibular system and its very widespread importance not just for oculomotor and postural control but for cognition and memory and the role of vestibular input to the basal ganglia, including its role in Parkinson's disease. His work had high impact: Google Scholar (31.12.2025) reports that he has had 15,941 citations with an H factor of 63. He has written four books and over 360 peer reviewed papers. He was a member of the editorial boards of a very large number of journals.

The breadth of his knowledge and contributions is amazing. He made original contributions both empirical and theoretical in so many areas, showing how vestibular input can impact many different human functions. Examples of his papers in his major research areas

- Galvanic stimulation for vestibular rehabilitation (4)
- The neural basis of tinnitus—neurochemistry and electrophysiology of tinnitus (5)
- Explore/evaluate therapeutic strategies for tinnitus (6)
- Vestibular- hippocampal function (spatial awareness, cognition, memory) (7–9), his most cited paper concerns the fact that vestibular loss causes hippocampal atrophy and impaired spatial memory (10)
- Vestibular basal ganglia connection (11) including vestibular function in Parkinson's disease (12)
- Aging and Alzheimer's disease (13)
- Pharmacology GABA, cannabinoids and the role transmitters in the restoration of the changes in neural activity which underlie vestibular compensation (14).

Paul attracted many graduate students, and he trained them well in how to do science and how to critically evaluate research and how to maintain very high standards for data acquisition and interpretation. He had numerous international collaborators in Japan, Europe, USA and Australia. He introduced many people to the role of vestibular input in such a huge variety of human behaviors.

Outside of the lab, Paul loved entertaining, cooking fiery curries, listening to a wide range of music, playing his vintage Gibson Les Paul guitar, and world travel. Paul will be missed by his many colleagues and international collaborators and a large number of people who he mentored. People particularly note his humility, his enthusiasm and his lifelong commitment to understanding the role of vestibular function in behavior. The attached photo captures the twinkle in his eye and his smile by which we will remember him.

## Conclusion

Vale Paul Smith—an excellent, scientist, collaborator, and friend.
